# Defining Yoga

**DOI:** 10.4103/0973-6131.43540

**Published:** 2008

**Authors:** H R Nagendra

**Affiliations:** Swami Vivekananda Yoga Anusandhana Samsthana (A Yoga University), No.9, Appajappa Agrahara, Chamarajpet, Bangalore - 560 018, India. Email: hrn@vyasa.org

Yoga is essentially practiced as ásanas, and ásanas are addressed as an alternate to exercises for a workout. To sweat out, a common feature of workouts, is evident in Yoga studios. No wonder that the Vikram Yoga is the hot spot. In India, traditionally Yoga is known as one of the six systems of Philosophy called Sat Darùanas – Nyaya, Vaisesika, Sankhya, Yoga, Pürva Mimamsa, and Uttara Mimamsa. Taking into consideration the very meaning of Yoga (Yujyate anena iti Yogah) [Slide 1]

**Slide 1:**
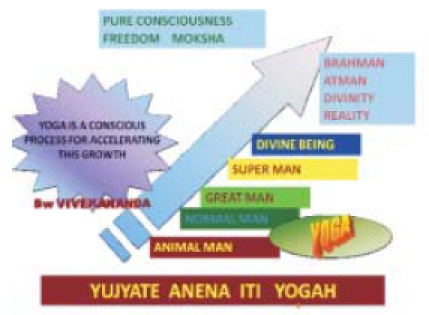
Concept of Yoga

Swami Vivekananda expanded the scope of Yoga to encompass all streams – Jòána, Bhakti, and Karma – also to lay the foundation for the four main streams of Yoga [Slide 2].

**Slide 2:**
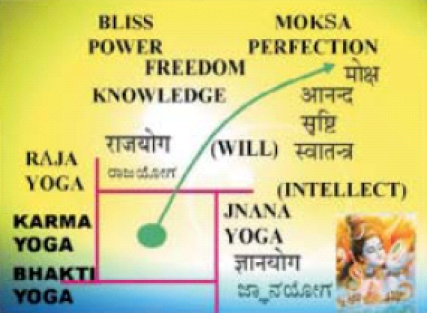
Streams of Yoga

And the purpose of all Yoga is to realize, be in tune, and ultimately merge with Reality; call it Perfection, Pure Consciousness, Parmatman, Nirvaïa as Buddha called it, Kaivalya as Pataòjali presented, Mokúa as Uttara Mèmamsa designates, or Reality which all scientists aim at, in all their research. He, without hair-splitting the semantic controversies, unifying them with an emphasis on the real spirit of Yoga as unification, to reach perfection, said that all paths mentioned above lead to the same goal in his famous proclamation, “Do it work or worship or psychic control or philosophy, by one or more or all of these and be free” [Slide 3].

**Slide 3:**
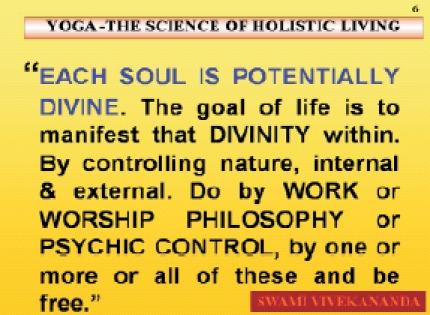
Each soul is potentially divine

That holistic definition of Yoga is most relevant to the modern society where the matter-based paradigm has become the truth of the times. Yoga postulates Consciousness-based paradigm and emphasizes that we are not robots governed by a set of physical laws driving us as machines. If we are more efficient than a robot sometimes or work haphazardly, we do it consciously with intelligence. This power of consciousness is featured by the freedom in all of us to choose – kartum, akartum, or anyatha va kartum – to do, not to do, or do it differently. Yoga is to enhance this freedom to choose the way to absolute freedom – freedom from all tensions and stresses, diseases, miseries – to move toward positive health, leading to perfect health.

Systematized and well-worked-out systems of Yoga, time tested for at least a few thousand years, aim at releasing us from all bondages – bondage of thoughts to begin with. We are bound by the thinking process right from the time we wake up till we sleep in the night, having no respite. This is the first bondage – says Pataòjali, the master of Yoga Darshana. Bondage of emotions – being tossed up and down in emotional upsurges, responding to the inner and outer inputs as if we are bound to respond like a stone falling down due to gravity determined by the three laws of classical mechanics. We realize in Bhakti Yoga that we can respond to inputs in our own chosen way – using the innate freedom for which we are known as human beings. In Karma Yoga again, we use this freedom to work without tension while discharging our duties, responsibilities, or even meeting our targets; who has pronounced the mandate that we should get tense when we need to meet a tough target? Working in relaxation, tension-free is the first lesson in Karma Yoga. Acting with a limited set of laws of the physical world while addressing more sophisticated multi-dimensional challenges of the modern era is called Ajòána, and to work with a higher set of laws is Jòána. Realization of the limitations of the matter-based paradigm is the first step. Jòána is featured by freedom. We move from one truth to the higher truth, from one level of freedom to the next level of higher freedom in our journey in Jòána Yoga. The ultimate is the release from even the bondage of the body and its laws.

Let me end with an incident in the life of Sri Ramakrishna. Mathura Babu was a brilliant modernized man, well-educated, and having the fullest faith in the matter-based paradigm. He said that the laws of the physical world are fixed and none can change the case of a stone falling down under the action of gravity. In his simplistic style, Sri Ramakrishna asked as to who made these laws? Mathura Babu said – none, no person, we call it nature. SR said, if that nature wants to change its laws, can it? No sir, straight came the answer. SR asked him to come next morning to the spot where there was a rose creeper. Next morning, Mathura Babu sees the usual red flowers in that creeper with a new white flower amidst!! Was Sri Ramakrishna working with the higher laws of creation – or of the creator? Mathura Babu was dumb-founded; all his belief got shattered about the faith in science. But the modern scientists, in their ignorance, would never accept the same. New generations of young scientists are coming up to fathom the higher levels of reality. Yoga raises us to higher levels of freedom with total understanding of the mechanisms and laws of creation.

To consider Yoga as yogasanas and yogasanas as physical exercises is to work with limited if not wrong knowledge of Yoga. Similarly, to use the terms “Yoga” and “meditation” is like saying Maths and arithmetic. Even the Yoga masters in India also have started using this phrase forgetting that meditation is the seventh limb of the Asûáñga yoga of Pataòjali. At least, shall we correct this wrong usage of the term “Yoga”?

